# Data and analysis script for infant and adult eye movement in an adapted ocular-motor serial reaction time task assessing procedural memory

**DOI:** 10.1016/j.dib.2020.105108

**Published:** 2020-01-08

**Authors:** Felix-Sebastian Koch, Anett Sundqvist, Ulrika Birberg Thornberg, Michael T. Ullman, Rachel Barr, Mary Rudner, Mikael Heimann

**Affiliations:** a Linköping University, Sweden; b Georgetown University, USA

**Keywords:** Procedural memory, Infancy, Memory development, Sequence learning, Serial reaction time task, Eye-tracking, Saccade latency extraction

## Abstract

This article provides a description of eye movement data collected during an ocular-motor serial reaction time task. Raw gaze data files for 63 infants and 24 adults along with the data processing and analysis script for extracting saccade latencies, summarizing participants’ performance, and testing statistical differences, are hosted on Open Science Framework (OSF). Files (in Matlab format) available for download allow for replication of the results reported in “Procedural memory in infancy: Evidence from implicit sequence learning in an eye-tracking paradigm” [1].

**Table:** Specifications Table

Subject	Developmental Neuroscience
Specific subject area	Infant cognitive development
Type of data	Individual gaze data files MATLAB ® code Summary tables
How data were acquired	Experiment run on Tobii ® T120 eye tracker with customized script in MATLAB ®
Data format	Raw Analysed Code
Parameters for data collection	Infants and adults were tested individually
Description of data collection	Eye gaze data were obtained while participants (infants and adults) performed an infant friendly adaptation of an ocular-motor version of the serial reaction time task.
Data source location	Infant and Child Lab, Department of Behavioural Sciences and Learning, Linköping University, Sweden
Data accessibility	Repository name: Open Science Framework Direct URL to data: https://osf.io/5npru/
Related research article	Author's name F.-S. Koch, A. Sundqvist, U. Birberg Thornberg, S. Nyberg, J.A.G. Lum, M. T. Ullman, R. Barr, M. Rudner, M. Heimann Title Procedural memory in infancy: Evidence from implicit sequence learning in an eye-tracking paradigm, Journal of Experimental Child Psychology 191, March 2020, 104733 DOI https://doi.org/10.1016/j.jecp.2019.104733

**Table:** 

**Value of the Data** • Infant and adult eye gaze data useful for reanalysis by other scholars. • Useful for examining human ocular-motor development from infancy to adulthood, in particular for meta-analysis. The ocular-motor system is essential for normal visual development and is affected in several diseases, e.g. nystagmus (infancy) and dementia (adults). • Useful for the development of future paradigms that access human ocular-motor development over the life-span, since the data suggests that similar processes are captured in both infants and adults. • Useful for future replication studies of the newly adapted task. • The serial reaction time task is a widely used task that for probing procedural learning that is generally based on manual responses and here ocular-motor responses for this task are examined.

## 1 Data

Data files are provided for participants (infants aged 9 month old: n = 63, n = 35 included in final analysis) and adults (mean age 24 years, n = 31) that performed the infant-friendly ocular-motor adapted serial reaction time task, reported on in Ref. [1]. Raw data files include the positions of the visual focus on the screen during the procedure. Furthermore, an analysis file is provided that extracts saccade latencies for all participants for all trials during the procedures, in addition to extracting further task-specific information. The script summarizes the data in a table that includes all participants. The script then continues and summarizes saccade latencies for blocks of trials, as is commonly done in serial reaction time task analysis [2], both for normalized saccade latencies and saccade latencies in milliseconds. It also performs statistical analysis on the data and presents the results from the analysis. All data and analysis files are in MATLAB ® format and are provided on Open Science Framework [3].

## 2 Experimental design, materials, and methods

The serial reaction time task (SRT) [4] is a well-established instrument for measuring procedural memory in humans and in its standard version is based on manual responses. The current data set includes saccade latencies instead of manual responses as reaction times.

During data collection, participants were seated individually (infants in their parent's lap) in front of a Tobii T120 eye tracker (Tobii AB, Stockholm, Sweden). The experiment was run through a custom script programmed in MATLAB (MathWorks Inc, Massachusetts, USA) version 2016b, that utilized Psychtoolbox, version 3.0.13 (Brainard, 1997; Kleiner, Brainard, & Pelli, 2007) and TobiiPro SDK (Tobii AB, Stockholm Sweden), version 1.1.0.21, for MATLAB. Participants viewed 125 images appearing consecutively in one of three on-screen positions (lower left corner, lower right corner, top center). Once participants fixated on the current image for 200 ms it disappeared and a new image at a new location appeared.

The MATLAB ® script “ocularMotorSRT_AnalysisScript.mat” loads all individual raw data files, pre-processes the gaze data, extracts saccade latencies and runs the statistical analysis (all files provided at https://osf.io/5npru/)[3].

### 2.1 Saccade latencies

The main variable of interest in this current experiment are saccade latencies in the eye movement data. In order to identify saccade latencies, the provided raw gaze data files are first preprocessed by the provided script (removing invalid data points and applying a moving average). Then the time window of interest is identified which is the time from 0.1 second to 2 seconds (or until a new trial was trigged if that was trigged before 2 seconds had passed) after an image appeared. Here eye movements faster than 35° per second are used to identify saccades. Saccade latencies are calculated if a gaze originated from the position of the previous image and ended in the position of the current image. Saccade latency is the time from when an image occurred on screen until the onset of the saccade. The script provides the identified saccade latencies for each trial for every participant in a data table (named “saccadeLatenciesAllTable”).

### 2.2 Serial reaction time task

As is common procedure in serial reaction time tasks [2], a mean for response times (here saccade latencies) is calculated for each block. In the current data set the 125 trials were subdivided into 5 blocks with 25 trials each. In block 1, 2, 3, and 5 the visual spatial order of image presentation on the screen followed a predefined order. In block 4 the order was pseudo-randomized. The provided script calculates the mean responds times per block for each participant (data table is named “srtDataTable”) and excludes participants that do not meet inclusion criteria. Data is summarized in tables with means per block for each participant (data table is named “srtDataTableValid”). In Ref. [1] data is reported that was first normalized by transformed saccade latencies to z-scores, before calculating the mean per block first. The script performs the transformations and presents the normalized data per individual. Additionally, the script summarizes the saccade latencies for each block without transforming the data. Analysis per block, normalized and untransformed, are presented in a data table created by the script provided.

### 2.3 Statistical analyses

Differences between blocks is the main variable of interest in the analysis of serial reaction time tasks. The script performs the mixed-design analysis of variance (ANOVA) reported in Ref. [1] based on transformed saccade latency scores. Additionally, the script performs analyses on untransformed scores [reported in supplementary material in 1]. The analyses are then displayed within MATLAB ®.
